# Assessing medication adherence and their associated factors amongst type-2 diabetes mellitus patients of Jazan Province, Saudi Arabia: A single-center, cross-sectional study.

**DOI:** 10.1016/j.jsps.2023.101896

**Published:** 2023-12-06

**Authors:** Amani Khardali, Nabeel Kashan Syed, Saad S. Alqahtani, Marwa Qadri, Abdulkarim M. Meraya, Norah Rajeh, Fatimah Aqeely, Sedan Alrajhi, Amnah Zanoom, Shahd Gunfuthi, Wahhaj Basudan, Thana K. Hakami, Mawada A. Abdelgadir

**Affiliations:** a Pharmacy Practice Research Unit, College of Pharmacy, Jazan University, Jizan - 45142, Jazan, Saudi Arabia; b Department of Clinical Pharmacy, College of Pharmacy, Jazan University, Jizan - 45142, Jazan, Saudi Arabia; c Department of Clinical Pharmacy, College of Pharmacy, King Khalid University, Abha, Saudi Arabia; d Department of Pharmacology and Toxicology, College of Pharmacy, Jazan University, Jazan-45142, Saudi Arabia; e Inflammation Pharmacology and Drug Discovery Unit, Medical Research Center, Jazan University, Jazan-45142, Saudi Arabia; f Jazan Endocrinology and Diabetes Centre, Jizan - 82723, Jazan, Saudi Arabia; g College of Pharmacy, Jazan University, Jizan - 45142, Saudi Arabia; h Faculty of Medicine, Jazan University, Jazan 45142, Saudi Arabia

**Keywords:** Type-2 diabetes mellitus, Medication adherence, Jazan Province, Saudi Arabia

## Abstract

**Background:**

The prevalence of type 2 diabetes mellitus (T2DM) globally is reaching epidemic proportions. By 2035, it is projected to increase to 417 million, which is of significant concern as T2DM represents the most oversized budget item in many healthcare systems, primarily due to the high rates of morbidity and mortality associated with the disease. The worldwide cost burden of T2DM has been inexorably growing. A key contributor to the remarkably high morbidity and mortality rates is poor glycemic control potentially associated with medication non-adherence.

**Aim:**

The present research’s main objective included assessing medication adherence among patients with T2DM in a single center in Jazan Province.

**Methods:**

Three hundred nine patients with T2DM participated in a cross-sectional survey over three months (September to November 2022). The study participants comprised 50.8 % (females) and 49.2 % (males), with a mean age of 44.12 years (SD ± 12.70). A 31-item self-report questionnaire was used for data collection.

**Results:**

Sixty-six percent of the sample were found to be adherent to their T2DM therapy. A positive association was noticed between the GMAS score and the participant’s age (r = 0.24; p < 0.01). The participants’ medication adherence was significantly associated with having age above 50 years (*χ^2^ =* 13.62; *p =* 0.001), residing in urban localities (*χ^2^ =* 21.37*; p < 0.001*), being married (*χ^2^ =* 12.80*; p =* 0.002), having glycated hemoglobin level more than 8 % (*χ^2^ =* 6.99*; p = 0.03*) and taking between one to three medications per day (*χ^2^ =* 17.63*; p < 0.001*).

**Conclusion:**

The majority of T2DM patients in the present study were found adherent to their anti-diabetic medications, particularly older patients. Future studies should focus on exploring the reasons for the reported high adherence among older patients and non-adherence among younger patients, as this could facilitate the development of a strategy to enhance adherence.

## 1 Introduction

Medication adherence is the extent to which patients obtain their medications as prescribed and instructed by their healthcare providers for the entire prescribed duration (Osterberg & Blaschke, 2005). The World Health Organization (WHO) mentioned that non-adherence to chronic disease medications is a rampant worldwide problem and that the prevalence of non-adherence is on a continuous rise (Sabate, 2003). Especially the secondary nonadherence, where the patients stop taking their medications after starting their therapy (Osterberg & Blaschke, 2005). Medication non-adherence is a challenging worldwide issue which not only results in the progression of the disease but also in increased cost of treatment (Alqarni et al., 2019; Naqvi et al., 2019). A report published by the WHO in 2005 mentioned that around 50 % of patients with chronic disease were found nonadherent to their medications in developed countries, and this could be even higher in underdeveloped countries (Sabate, 2005). Indeed, medication adherence is much better in patients with acute conditions compared to chronic conditions, such as diabetes mellitus (Lehmann et al. 2014; Lam and Fresco 2015).

The International Diabetes Federation (IDF) has reported that the KSA is the seventh county worldwide with the highest prevalence of diabetes (17.7 %) (International Diabetes Federation 2021). More particularly in the Jazan region, Saudi Arabia, the prevalence of diabetes was 12.3 % (Bani, 2015). Type 2 diabetes mellitus (T2DM) is one of the most prevalent metabolic disorders (reed, 2021). Patterns and trends have revealed the Middle East to be among the countries with the highest increase in patients with T2DM (El-Kebbi et al. 2021). According to a systemic review conducted in 2020, 48 million people in the Middle East had T2DM (Ewid et al., 2023). Unfortunately, five of the top ten countries having the highest prevalence of diabetes are from the Middle East, and it is estimated that there might be a 90 % rise in the growth of the disease by 2030 (Bener et al., 2009, Shaw et al., 2010). The Kingdom of Saudi Arabia (KSA) is ranked third in the Middle East with a prevalence of 21.4 % after Kuwait (21.9 %) and the United Arab Emirates (21.6 %)(Kalan Farmanfarma et al., 2020). A recent study reported that obesity and other non-communicable diseases were among the potential risk factors for the reported high prevalence percentage among the population in the Gulf region, especially in Saudi Arabia(Okati-Aliabad et al., 2022).

Indeed, managing diabetes mellitus usually requires multifactorial approaches, including long-term pharmacological treatments, physical activities, and weight management (Davies et al. 2022). Nevertheless, some of patients do not achieve the required benefit of these treatment approaches due to the lack of the appropriate medication adherence (Brown and Bussell 2011). The most worrying part of the non-adherence is the increasing unabated complications (AlDawish et al., 2016, Alatawi et al., 2016). T2DM is associated with microvascular complications like diabetic nephropathy, diabetic neuropathy, and diabetic retinopathy. In Saudi Arabia, it is estimated that diabetic retinopathy could affect around 31 % of patients with diabetes. The national incidence of diabetic nephropathy among patients with end-stage renal failure in Saudi Arabia was reported to be approximately 50 % (Alatawi et al., 2016). T2DM could also be associated with macro-vascular complications like cardiovascular diseases, such as coronary heart disease and stroke (Alatawi et al., 2016; Wilhide et al., 2016). The risk of these complications and comorbidities could be drastically reduced if the patients adhered to their anti-diabetic medications while adopting a healthier lifestyle (AlQarni et al., 2019, Alqarni et al., 2018, Khan et al., 2012). Furthermore, anti-diabetic medications have the potential to control blood glucose levels from fluctuating to either hyperglycemia or hypoglycemia, and these fluctuations negatively impact the patient’s health and well-being (AlQarni et al., 2019, Alqarni et al., 2018).

Suboptimal adherence to anti-diabetic medication was reported in previous studies conducted in different regions of Saudi Arabia (e.g., Al Hasa, Al-Khobar and Bisha) (AlQarni et al., 2019, Alqarni et al., 2018, Khan et al., 2012). However, to the very best of the author’s knowledge, there is no information on the magnitude of non-adherence of patients with diabetes in the Jazan region of Saudi Arabia. Therefore, this study was conducted to assess medication adherence and its associated factors among patients of Jizan city, located in the southern Province of Saudi Arabia, using the General Medication Adherence Scale (GMAS) (Naqvi et al., 2020).

## 2 Methods

### 2.1 Study design, setting, and population

The current observational cross-sectional study was conducted for a duration of three months between 01 September 2022 and 30 November 2022 at the Diabetic & Endocrinology Centre of Jazan Hospital, Jazan City, Jazan Province, Saudi Arabia. The researcher invited patients having outpatient appointments at the endocrine clinics of the Diabetic Centre to participate in the study. Convenience sampling was used for participant selection and recruitment. All the study participants completed a 31-item self‐administered questionnaire.

### 2.2 Inclusion and exclusion criteria

Participants included in the current study were required to be (1) Male and female patients over 18 years of age, (2) diagnosed with T2DM and cross-verified either by medical records or lab reports, (3) have complete medical records, (4) prescribed T2DM medications, (5) agreeable to participate in the current study and willing to complete the survey. Those participants who did not fulfil the inclusion criteria were excluded from the current study.

### 2.3 Data collection

Two different approaches were used to collect data in the current study. The first approach included a self-administered questionnaire with three sections: demographic, clinical information, and GMAS sections. At the same time, the second approach included the review of the patient’s medical records.

A list of eligible patients based on the inclusion criteria was prepared by the research team (A.K, N.R, F.A, S.A, A.Z, S.G, W.B, and T.H), then the patient’s physician was asked to introduce the study to the patients and handled them the participant information sheet. After the patients' clinic visits, they were approached by the research team and were given the opportunity to ask further about the study. If the patients agreed to participate in the current study, they were given a choice to complete the study questionnaire on the researcher's iPad® device (in a private and quiet area at the center) or submit the study questionnaire to them, and they were asked to complete the self-administered questionnaire online at their convenience. The participants were requested to complete all the questions across all questionnaire sections. An average duration of 10 min was needed to complete the questionnaire. The return of the filled questionnaire to the research team was considered implied consent. A follow-up notification was sent to non-responders after 2 weeks from their appointment.

Furthermore, the medical records of the patients who agreed to participate were screened to check and complete other demographic and clinical information.

### 2.4 Sample size

Sampsize Calculator (https://sampsize.sourceforge.net/iface/) was used to calculate the current study’s sample size. Based on a prevalence rate of 12.3 %, as reported by a similar survey from Jazan (Bani, 2015), along with a confidence interval of 95 % and a precision of 5 %, we estimated the sample size for the current research to be 166. Furthermore, the research team considered the addition of 10 % to allow for withdrawal from the study; therefore, an estimated sample of 183 patients was required for this study. Out of 350 patients approached, 309 (88 %) patients agreed to participate and returned the filled questionnaire, which exceeded the required sample size.

## 3 Measures

### 3.1 Participants’ socio-demographics

The current study participants recorded their gender, nationality, age group *(in years)*, age *(in years)*, location of residence, marital status, employment status, glycated haemoglobin levels, current anti-diabetic medications, co-morbidities, and quantity of current medications.

### 3.2 General medication adherence scale (GMAS)

Medication adherence in the current study was measured by the Arabic version of the 11-item GMAS (Naqvi et al., 2020). GMAS comprises three sections; the first section consists of five items assessing the patient’s behavior-related non-adherence (PBNA); questions 1–5, and the second section includes four items assessing the additional disease and pill burden-related non-adherence (ADPB); questions 6–9. The last section consists of two items evaluating the patients’ cost-related non-adherence (CRNA); questions 10–11 (Naqvi et al., 2020). The responses of 11-item GMAS are recorded on a four-point Likert scale from 0 (always) to 3 (never). The 11-item GMAS score ranges from 0 to 33, where higher scores denote greater medication adherence. The patient’s score is categorized as adherent if the total score is greater than 27 (or) non-adherent if the total score is less than 26 (Naqvi et al., 2020).

### 3.3 Ethical approval

The Standing Committee for Scientific Research at Jazan University, Saudi Arabia (HAPO-10-Z-001) approved the protocol of this study (Approval Reference No: REC-44/07/500). Jazan Health Ethics Committee, Ministry of Health Affairs, Jazan Province, Saudi Arabia, also granted its ethical approval for the conduct of this study (Approval Reference No: 22105). Participation in the current research was voluntary, and the participants could withdraw from the study at any given time without any potential consequences. Participants were also informed beforehand that the data would only be used for scientific purposes and were assured that it was strictly confidential.

### 3.4 Data analysis

The statistical software IBM SPSS (Statistical Package for Social Sciences, SPSS Inc. Chicago, IL, USA) version 23 was used for the data analysis of the current study. The *Google Forms* data were transcribed into an Excel sheet and later exported to SPSS. Data were coded to ensure confidentiality for all participants. The participants’ socio-demographics were expressed as frequencies, total percentages, means, and standard deviations. For assessing any statistically significant associations between variables, crosstabulations with Pearson’s chi-square test were used. Whilst for variables having a count less than 5, Fisher’s Exact test was used. A multivariate binary logistic regression model examined any significant associations between explanatory and outcome variables. The independent variables included in the model were age group, location, marital status, occupation status, glycated Hemoglobin levels, anti-diabetic medications, co-morbidity, and quantity of daily medication. A significant association was reported in *p* < 0.05.

## 4 Results

A total of 309 patients completed the questionnaire, with an age range between 26 and 68 years old. Table 1 illustrates the participants’ socio-demographic and clinical characteristics of study patients.

**Table 1:** Socio-demographic and clinical characteristics of respondents (N = 309):

Variable	Options	Frequency (n)	%
Gender	Male	152	49.2
	Female	157	50.8
Nationality	Saudi	309	100
Age group *(in years)*	18–29	56	18.1
	30–49	112	36.2
	> 50	141	45.6
Exact age *(in years)*	Mean 44.12	**SD** ± 12.70	
Location of current residence	Urban	178	57.6
	Rural	131	42.4
Marital Status	Unmarried / Single	62	20.1
	Married	213	68.9
	Divorced / Widowed	34	11.0
Employment Status	Student	38	12.3
	Employed	104	33.7
	Retired	44	14.2
	Unemployed	123	39.8
Glycated Hemoglobin Levels	≤ 7 %	35	11.3
	7 % − 8 %	88	28.5
	≥ 8 %	186	60.2
Current Anti-Diabetic Medications	Oral Hypoglycemic Agents (OPA) Only	65	21.0
	Oral Hypoglycemic Agents & Insulin	167	54.0
	Insulin Only	77	24.9
Co-morbidities	Yes	147	47.6
	No	162	52.4
Quantity of current medications	1–3	122	39.5
	4–6	110	35.6
	7–9	63	20.4
	> 10	14	4.5

The percentage of female participants (n = 157; 50.8 %) was slightly higher than their male counterparts. The nationality of all the participants was Saudi. Regarding the age group of the study sample, it was noted that almost half (n = 141; 45.6 %) were above 50 years, with a mean age of 44.12 years (SD ± 12.70). Over half of the participants (n = 178; 57.6 %) resided in urban localities. A high percentage of the sample (n = 213; 68.9 %) were married. Regarding the employment status of the study sample, it was noticed that more than half (n = 167; 53.6 %) were either unemployed (or) retired at the time of the study. Most participants (n = 186; 60.2 %) had a Glycated hemoglobin level of more than 8. With regards to the current anti-diabetic medications, it was observed that more than half of the study sample (n = 167; 54.2 %) were taking oral hypoglycemic agents along with insulin. Around half of the participants (n = 147; 47.6 %) had co-morbidities. More than half of the study participants (n = 185; 59.9 %) took more than four drugs.

Sixty-six percent of patients were found adherent to their anti-diabetic medications, while 34 % were found nonadherent. It was seen in the current study that nearly a quarter (n = 81; 26.2 %) of the study sample reported that they always/mostly find it difficult to buy medications because of their cost (n = 5; 1.6 %) of the study participants missed their medicines due to progression of the disease and addition of new drugs. More than half of the participants never had difficulty in remembering to take their medication, never forgot to take their medication owing to a busy schedule, never discontinued their medication after feeling better, never stopped taking medication after observing adverse effects, never stopped taking medicines without informing their doctor, never discontinued their medication due to additional disease, never found taking their medications a hassle due to complexity of their medication regime, never altered their dose, medication frequency by their own, and never discontinued their medicines as they were not worthy of your money. Table 2 depicts further details on the descriptive results of the GMAS.

**Table 2:** Descriptive of the participants’ responses to the Arabic version of the GMAS.

***Arabic version of GMAS***	***Always n(%)***	***Mostly n(%)***	***Sometimes n(%)***	***Never n(%)***
1.Do you have difficulty in remembering to take your medications?	7 (2.3 %)	23 (7.4 %)	104 (33.7 %)	175 (56.6 %)
2. Do you forget to take your medication due to your busy schedule, traveling, meeting, events at home, party, marriage, religious celebrations, etc.?	13 (4.2 %)	28 (9.1 %)	113 (36.6 %)	155 (50.2 %)
3. Do you discontinue your medication when you feel well?	14 (4.5 %)	25 (8.1 %)	49 (15.9 %)	221 (71.5 %)
4. Do you stop taking medications when you feel adverse effects such as gastric discomfort, etc.?	12 (3.9 %)	30 (9.7 %)	84 (27.2 %)	183 (59.2 %)
5. Do you stop taking medications without informing the doctor?	10 (3.2 %)	11 (3.6 %)	58 (18.8 %)	230 (74.4 %)
6 Do you discontinue your medicines due to other medicines that you have to take for your additional disease?	6 (1.9 %)	9 (2.9 %)	41 (13.3 %)	253 (81.9 %)
7. Do you find it is a hassle to remember your medications due to medication regime complexity?	14 (4.5 %)	23 (7.4 %)	66 (21.4 %)	206 (66.7 %)
8. During the last month, had there been any occasion when you missed your medicines due to progression of disease and addition of new medicines?	5 (1.6 %)	21 (6.8 %)	76 (24.6 %)	207 (67.0 %)
9. Do you alter medication regimen, dose, and frequency by yourself?	16 (5.2 %)	33 (10.7 %)	80 (25.9 %)	185 (58.2 %)
10. Do you discontinue these medications because they are not worth the money you spent on them?	5 (1.6 %)	9 (2.9 %)	24 (7.8 %)	271 (87.7 %)
11. Do you find it difficult to buy your medicines because they are expensive?	39 (12.6 %)	42 (13.6 %)	72 (23.3 %)	156 (50.5 %)

**Fig. 1:**
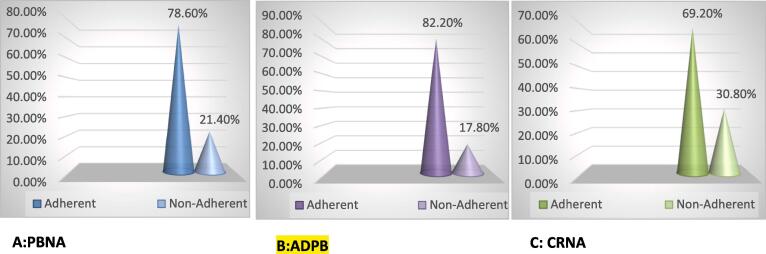
Adherence-related factors; A PBNA, Patient behaviour related non-adherence; B ADPB Additional disease and pill burden related non-adherence; C, CRNA, Cost related non-adherence.

The factors related to non-adherence, which included patient’s behaviour (intentional and unintentional), comorbidity and polypharmacy, and cost-related factors were also assessed using GMAS. Three-quarters of the patients (78.6 %) were found to be adherent to their anti-diabetic medications and a very low percentage of the patients (21.4 %) were non-adherent to their therapy owing to their behavior. The majority of patients (82.2 %) were found adherent to their anti-diabetic medications, while only a meager percentage (17.8 %) were non-adherent to their medications due to co-morbidities and pill burden. It is observed that more than half of the participants (69.2 %) were adherent to their anti-diabetic medications, and a significant percentage (30.8 %) were found to be non-adherent because of financial constraints. See Fig. 1(A-C).

Table 3 illustrates the association between participants’ sample characteristics and different adherence categories. The participants’ medication adherence was significantly associated with being above the age of 50 years (*χ^2^ =* 13.62; *p =* 0.001), residing in urban localities (*χ^2^ =* 21.37*; p < 0.001*), being married (*χ^2^ =* 12.80*; p =* 0.002), having glycated hemoglobin level more than 8 % (*χ^2^ =* 6.99*; p = 0.03*) and taking between one to three medications per day (*χ^2^ =* 17.63*; p < 0.001*). Participants’ gender, occupation, and current anti-diabetic medications were not significantly associated with their medication adherence.

**Table 3:** Crosstabulations between sample characteristics and adherence categories:

**Variable**	**Non – Adherent**	**Adherent**	**Total n (%)**	**(Chi-square) χ^2^**	**p-value**
***Gender***					
Male	55 (51.9 %)	97 (47.8 %)	152 (49.2 %)	0.47	0.49
Female	51 (48.1 %)	106 (52.2 %)	157 (50.8 %)		
***Age Group***					
18–29	17 (16.0 %)	39 (19.2 %)	56 (18.1 %)	13.62	0.001
30–49	53 (50.0 %)	59 (29.1 %)	112 (36.2 %)		
> 50	36 (34.0 %)	105 (51.7 %)	141 (45.6 %)		
***Location***					
Urban	42 (39.6 %)	136 (67.0 %)	178 (57.6 %)	21.37	*p <* 0.001
Rural	64 (60.4 %)	67 (33.0 %)	131 (42.4 %)		
***Occupation***					
Student	8 (7.5 %)	30 (14.8 %)	38 (12.3 %)	4.46	0.22
Employed	34 (32.1 %)	70 (34.5 %)	104 (33.7 %)		
Retired	18 (17.0 %)	26 (12.8 %)	44 (14.2 %)		
Unemployed	46 (43.4 %)	77 (37.9 %)	123 (39.8 %)		
***Marital Status***					
Single	33 (31.1 %)	29 (14.3 %)	62 (20.1 %)	12.90	0.002
Married	61 (57.5 %)	152 (74.9 %)	213 (68.9 %)		
Divorced /Widower	12 (11.3 %)	22 (10.8 %)	34 (11.0 %)		
***Glycated Hemoglobin levels***					
< 7 %	15 (14.2)	20 (9.9)	35 (11.3)	6.70	0.03
7 % − 8 %	38 (35.8)	50 (24.6)	88 (28.5)		
> 8 %	53 (50.0)	133 (65.5)	186 (60.2)		
***Current Anti-diabetic medications***					
OPA only	19 (17.9 %)	46 (22.7 %)	65 (21.0 %)	2.61	0.27
OPA & Insulin	64 (60.4 %)	103 (50.7 %)	167 (54.0 %)		
Insulin only	23 (21.7 %)	54 (26.6 %)	77 (24.9 %)		
***Co-morbidities***					
Yes	69 (65.1 %)	78 (38.4 %)	147 (47.6 %)	21.37	*p <* 0.001
No	37 (34.9 %)	125 (61.6 %)	162 (52.4 %)		
***Quantity of current daily medication***					
1–3	26 (24.5 %)	97 (47.3 %)	122 (39.5 %)	17.63	0.001
4–6	49 (46.2 %)	61 (30.0 %)	110 (35.6 %)		
7–9	23 (21.7 %)	40 (19.7 %)	63 (20.4 %)		
> 10	8 (7.5 %)	6 (3.0 %)	14 (4.5 %)		

### 4.1 Odds of being medication adherent

The current research’s outcome variable is binary (i.e., medication adherent/ non-adherent). Hence, a multi-variable binary logistic regression was used to assess possible associations between the outcome variable and the study participants’ socio-demographics and clinical variables. Table 4 depicts the results of the multi-variable binary logistic regression. The participants aged 30–49 years were less likely to be medication adherent in comparison to those above the age of 50 years (AOR: 0.24; CI 95 %:0.11–0.51; *p* < 0.001). Single/unmarried individuals were less likely to be medication adherent than those who were divorced/widowed (AOR: 0.21; CI 95 %:0.06–0.76; *p* < 0.05). Regarding the participant’s current location, it was noticed that those residing in urban localities were more likely to be medication adherent than those who resided in rural localities (AOR: 2.28; CI 95 %:1.27–4.09; *p* < 0.01). Students were more likely to be medication adherent than those unemployed participants (AOR: 5.24; CI 95 %: 1.17–23.47; *p* < 0.05). Also, it was noticed that those participants with co-morbidities were less likely to be medication adherent as compared with those with no co-morbidities (AOR:0.38; CI 95 %:0.19–0.74; *p* < 0.01). With regards to the quantity of daily medication, it was noticed that those participants taking medications in the range of one to three (i.e., <4 medicines/day) were more likely to be medication adherent than those taking more than ten medicines daily (AOR: 7.22; CI 95 %: 1.60–32.51; *p* < 0.05).

**Table 4:** Odds of medication adherence with participants’ socio-demographics.

***Determinant***	***Adjusted Odds Ratio (AOR)***	***95 % C.I.***		*p-*value
		Lower	Upper	
***Gender***				
Male	0.62	0.32	1.19	0.15
Female				*Ref*
***Age Group***				0.001
18–29	0.37	0.08	1.65	0.19
30–49	0.24	0.11	0.51	*p <* 0.001
> 50				*Ref*
***Location***				
Urban	2.28	1.27	4.09	0.006
Rural				*Ref*
***Marital Status***				*0.002*
Single	0.21	0.06	0.76	0.02
Married	1.26	0.50	3.19	0.63
Divorced / Widower				*Ref*
***Occupation***				0.05
Student	5.24	1.17	23.47	0.03
Employed	1.99	0.91	4.38	0.09
Retired	0.85	0.33	2.18	0.73
Unemployed				*Ref*
***HbA1C***				0.24
< 7 %	0.48	0.20	1.19	0.11
7 % − 8 %	0.72	0.38	1.37	0.32
> 8 %				*Ref*
***Current Anti-Diabetic Medication***				0.85
OPA only	1.35	0.47	3.93	0.58
OPA & Insulin	1.27	0.49	3.26	0.63
Insulin only				*Ref*
***Co-morbidities***				
Yes	0.38	0.19	0.74	0.005
No				*Ref*
***Quantity of Daily Medication***				0.02
1–3	7.22	1.60	32.51	0.01
4–6	2.52	0.65	9.74	0.18
7–9	3.24	0.84	12.45	0.09
Greater than 10				*Ref*

C.I. = Confidence Interval: Variables included in the multi-variable binary logistic regression model were participants’ gender,

age group, and location. Marital status, occupation, glycated hemoglobin levels, current diabetic medication, co-morbidities, and.

quantity of daily medication. Medication adherence was the outcome variable.

## 5 Discussion

The assessment of adherence is cumbersome and primarily dependent on the patient’s intentional and unintentional behaviors (Osterberg and Blaschke, 2005). Adherence to anti-diabetic regimens is essential for maintaining optimal blood glucose levels. Consequently, failure to adhere could lead to a major problem. Therefore, in the current study, anti-diabetic medication adherence and its potential association with blood glucose levels were assessed among T2DM patients in a single center in Jazan Province, Saudi Arabia.

Our study revealed that the majority of patients with T2DM were adherent to their medications. Similar to Naqvi et al. (2020), this study used a cut-off score of greater than 27 of the total GMAS score to categorize the patients as being medication adherent and any score lesser than 26 as being medication non-adherent. Based on these scores, the prevalence of medication adherence was 66 %. However, the previous studies conducted in the other regions of KSA reported a high percentage of non-adherence among their participated patients with T2DM (Khan et al., 2012, AlQarni et al., 2019, Alhazmi et al., 2017). A study conducted in the Al Hasa district, KSA, assessing different factors contributing to non-adherence among people with T2DM, reported the prevalence of therapeutic non-adherence to be 67.9 % (Khan et al., 2012). In addition, non-adherence among patients with T2DM was reported in different countries in the Middle East, such as Jorden (58 %) (Jarab et al., 2014, Al-Qerem, et al., 2022), Palestine (52.7 %) (Almadhoun et al 2018), Qater (73 %) (Jaam et al., 2018), and United Arab Emirates (61.7 %) (Ashames et al., 2021). Indeed, the data from the current study did not allow for further investigations; therefore, another qualitative study was planned and conducted to provide more explanation regarding the high reported adherence percentage among patients with T2DM in Jazan. However, Some of the possible reasons for the increased adherence to anti-diabetic medications in the present study could be due to the effective implementation of Vision, 2030 guidelines, the most widely accepted and forward-thinking national strategic plan. One of the primary objectives of the Vision, 2030 guidelines is to achieve economic as well as health prosperity in KSA by the year 2030, that which has recognized the complete eradication of health discrepancies in chronic disease patients as an utmost priority of public health (Chowdhury et al., 2021) ‘Vision, 2030 - Quality of Life (QOL) program’, has targeted diabetes which is a critical chronic disease condition. The foremost strategies of this initiative in controlling and preventing diabetes include improvement of the acceptability and ease of access to treatment as well as preventative healthcare services for patients with diabetes across different regions of KSA (Itumalla et al.,2021).

KSA currently provides free health care to all its citizens and expatriates working in the public sector, which is done mainly via the Ministry of Health (MOH) as well as through other governmental health facilities (Watson et al.2008). The MOH manages primary healthcare centers (PHCs). This nationwide system provides free healthcare services for Saudi citizens and expatriates needing simple medical procedures and those suffering from non-communicable diseases (Alanazi et al.,2023). Free access to healthcare could be one of the potential reasons for improved medication adherence. A study conducted in different primary, secondary, and tertiary healthcare settings in Islamabad, Pakistan, for studying non-adherence to prescribed antihypertensive medications reported that access to free healthcare was a potential reason for improved medication adherence (Mahmood et al.,2020).

Regarding the different socio-demographic characteristics impacting medication adherence, it was noticed that the participants’ gender did not have any significant association with medication adherence. This finding is consistent with AlQarni et al. (2019), who also reported that participants’ gender not to be significantly associated with medication adherence to anti-diabetic medications. Our findings contrast the results of Khan et al. (2012) and Elsous et al. (2017), who reported that gender had a significant association with non-adherence and that female patients were more adherent than male patients. Consistent with a recent study conducted in Jordan, despite no significant association between gender and medication adherence, the findings revealed that female patients reported better adherence than male patients (106 vs. 97 patients) (Al-Qerem et al., 2022).

Similar to the previous study, the patient’s age group in the present sample was significantly associated with medication adherence (*p <* 0.01) (Sah et al., 2021). In contrast to our findings, Khan et al. (2012) and Sakya et al. (2023) reported that medication adherence has no significant association with participants’ age. Pearson’s correlation test also showed that age positively correlated with adherence to anti-diabetic medications (r = 0.24; *p <* 0.01). This finding is consistent with AlQarni et al. (2019), who also reported that medication adherence positively correlated with participants’ age. A recent study conducted to assess medication adherence among geriatric patients with chronic diseases reported a high adherence percentage (82 %) (Punnapurath et al., 2021). This could be attributed to the fact that older patients have a longer experience with medication therapies, which might result in enhancing awareness regarding the importance of medication adherence. The present study did not find patients’ occupation to impact their adherence to anti-diabetic medications, as was evident from a non-significant association between the two variables. Similar findings were reported by Shakya et al. (2023), who also did not observe any significant association between medication adherence and participants’ occupation. In contrast, Alqarni et al. (2018) reported that the occupation of the study participants was significantly associated with their medication adherence.

Three-quarters (74.9 %) of the adherent patients in the current study were found to be married, thus yielding a significant association between the two variables (*p <* 0.01). Alqarni et al. (2018) also reported that only (36.4 %) of their sample with high adherence to anti-diabetic medications were married (*p >* 0.05). In contrast, Nazir et al., 2016, Wu and Liu, 2016, Gu et al., 2017, Lee et al., 2017, Mentock et al., 2017, Abdullah et al., 2019 reported that participants’ marital status not to be significantly associated with the medication adherence. In terms of the glycated hemoglobin levels (HbA1C) of the current study, patients had a significant association with their medication adherence (*p <* 0.05). These findings are consistent with a study conducted in Singapore assessing medication adherence in T2DM patients, which reported that patients’ glycated hemoglobin levels are significantly associated with medication adherence (Lee et al., 2017). Zhang et al. and Abdullah et al. reported similar findings wherein participants’ medication adherence and HbA1c levels were significantly associated (Abdullah et al., 2019, Zhang et al., 2021). Patients’ current anti-diabetic medications did not significantly impact their medication adherence. These findings are consistent with Shakya et al. (2023), who also reported that patients’ medication adherence was not associated with anti-diabetic medications. Conversely, Khan et al. reported dissimilar findings where the patients’ polypharmacy was significantly associated with their medication adherence (Khan et al., 2012). Patients without co-morbidities were more adherent to their anti-diabetic medications than those with co-morbid conditions (*p <* 0.001). Co-morbidities have previously been highlighted as a significant impediment to medication adherence (Al-Saeedi et al., 2002, Newman-Casey et al., 2015, Jin et al., 2016, Yap et al., 2016). More than three-quarters of the sample taking less than six medications were found to be more adherent to their medications than those taking more than seven medications (*p <* 0.01). Similar findings were also reported by Horii et al. (2019), Jabar et al (2014), and Al-Qazaz et al. (2011), who reported medication adherence to be significantly associated with the number of simultaneous medications the patients took.

There are a number of limitations in this study, including the study duration and the cross-sectional nature, which would prevent causality assessment. The patients were also recruited from only one center in the Jazan region, which made the generalizability of the study findings to the whole region and different regions in KSA very difficult. In addition, the participating patients were only Saudi citizens; the non-Saudi T2DM patients living in the regions might provide valuable insight into a comprehensive understanding of the pattern of medication adherence, especially related to the factors associated with non-adherence. However, the Diabetic & Endocrinology Centre of Jazan Hospital is the only specialized diabetes center in the Jazan region that has patients from across Jazan region, and the majority of patients treated in this center were Saudi citizens and non-Saudi who are eligible for free treatment based on the Saudi Ministry of Health criterion. Convenience sampling would prevent the sample from being representative. Nevertheless, the number of responders exceeded the required sample size for this study. Finally, a self-administered questionnaire is generally subject to recall bias and social desirability which might have impacted the quality of the collected data.

## 6 Implications and directions for future research

There are ongoing public health reforms in Saudi Arabia to minimize unnecessary expenditure on healthcare services and maximize the outcomes of healthcare services provided to the Citizens and Residents of the KSA. Enhancing adherence to anti-diabetic medications would have a significant impact on health economics, patient health outcomes, and clinical practice. Several measures and strategies were previously reported that might enhance patient adherence to the medications, such as simplifying treatment regimens, involving healthcare professionals (pharmacists) in patient medication management, and educating patients about their condition and medications (Jarab et al., 2014, AlQarni et al., 2019, Naqvi et al., 2020, Al-Qerem, et al., 2022). However, choosing the appropriate strategy could differ based on the needs of the target populations and their problems. The findings from this study warrant further investigation to explore the barriers to non-adherence in patients aged between 30 and 49 years old and facilitators to medication adherence among older patients to develop suitable strategies that would improve patient's health outcomes and reduce the economic burden associated with medication non-adherence in the healthcare system in the KSA.

## 7 Conclusions

Medication adherence for T2DM patients is an essential factor in helping manage the condition and preventing further associated complications. This study found that T2DM patients in Jazan are more likely to be adherent to their anti-diabetic medications. Factors significantly impacting adherence were age, marital status, geographic location, poor glycemic control, polypharmacy, and co-morbidities. Finally, additional research is needed to explore medication adherence among patients with T2DM to identify factors that could contribute to non-adherence to plan successful strategies to enhance medication adherence in Jazan, Saudi Arabia.

## Declaration of Competing Interest

The authors declare that they have no known competing financial interests or personal relationships that could have appeared to influence the work reported in this paper.
